# Pregnancy and Dry Eye Syndrome: A Review for Clinical Practice

**DOI:** 10.3390/ijms26209990

**Published:** 2025-10-14

**Authors:** Marta Jaruchowska, Joanna Przybek-Skrzypecka, Janusz Skrzypecki

**Affiliations:** 1 ISPL Marta Jaruchowska, 13-200 Działdowo, Poland; jaruchowska.firma@gmail.com; 2 Department of Ophthalmology, Medical University of Warsaw, 02-097 Warsaw, Poland; 3 SPKSO Ophthalmic University Hospital, 03-709 Warsaw, Poland; 4 Department of Experimental Physiology and Pathophysiology, Medical University of Warsaw, 02-097 Warsaw, Poland

**Keywords:** dry eye syndrome, pregnancy, hormone, tear film

## Abstract

Pregnancy is a unique physiological state associated with profound hormonal, metabolic, and immunological changes that may affect ocular surface homeostasis. Among these changes, fluctuations in estrogen and progesterone are the most consistent factors contributing to the development or exacerbation of dry eye syndrome (DES), although insulin resistance and maternal immune tolerance may also play a role in modulating disease severity. Current evidence suggests that DES can affect between 20% and 50% of pregnant women, most commonly during the second and third trimesters. Symptoms are often mild and self-limiting, but they can negatively impact quality of life and adherence to prenatal care. Importantly, however, current consensus reports such as TFOS DEWS III emphasize that pregnancy has not been established as a confirmed risk factor for DES and the evidence remains inconclusive despite the biological plausibility of hormonal influences. This review summarizes the pathophysiological mechanisms underlying DES in pregnancy, highlights diagnostic approaches and safe management strategies tailored for pregnant women, and emphasizes the importance of awareness among obstetricians and ophthalmologists. Greater recognition of DES in pregnancy may improve patient comfort and prevent long-term ocular complications.

## 1. Introduction

**Figure 1:**
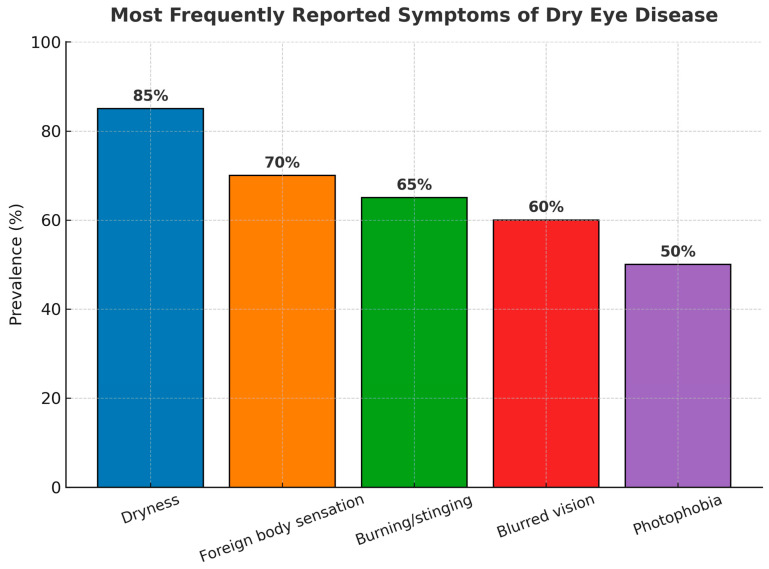
The most frequently reported symptoms of DES. Ocular dryness, foreign body sensation, and burning/stinging are the most common, followed by eye fatigue, photophobia, and fluctuating vision. Percentages are based on data summarized in the TFOS DEWS II Definition and Classification Report [1].

Dry eye syndrome (DES), also known as keratoconjunctivitis sicca, is a common ocular disease characterized by reduced tear production, excessive tear evaporation, or an imbalance in the tear film’s composition [1]. Patients most often report ocular discomfort, dryness, burning, foreign body sensation, or grittiness, with the most frequently described symptoms summarized in Figure 1.

The prevalence of DES varies widely, influenced by age, gender, environmental exposure, and both genetic and lifestyle factors [1,2,3,4,5]. In developed countries, it affects approximately 8.4% of younger individuals and up to 19.0% of those over 60 years old, depending on associated risk factors and research methodology [4,6,7,8,9]. DES becomes more common after the age of 40 in both sexes, with prevalence increasing progressively with age. In women, the rise is particularly pronounced after the age of 50, which may be related to hormonal changes associated with ovarian senescence [10]. The postmenopausal decline in estrogen and androgen levels destabilizes the tear film and impairs meibomian gland function, thereby contributing to the higher disease burden observed in this population [5,11,12,13]. However, the association between menopause and DES remains uncertain [10]. A careful review is needed to determine whether the higher prevalence of DES in postmenopausal women is due to menopause itself or simply reflects aging and age-related comorbidities. While much of the literature focuses on postmenopausal women, physiological and hormonal changes during pregnancy may also initiate or exacerbate DES. Although the available data are limited, current studies suggest that it can affect even 20–50% of pregnant women, most commonly during the second and third trimesters [14,15,16]. Pregnancy induces significant hormonal shifts—including changes in estrogen, progesterone, and testosterone—that affect tear film stability, ocular surface homeostasis, and Meibomian gland function (MDG) [17].

Beyond reproductive status, the pathogenesis of DES reflects a multifactorial interplay between intrinsic and extrinsic risk factors [1,2,18]. Intrinsic factors include age over 50, female gender, autoimmune and systemic diseases (e.g., Sjögren’s syndrome, thyroid dysfunction, rheumatoid arthritis, lupus, diabetes), neurological disorders (e.g., Parkinson’s disease, sensory neuropathies), and MGD [1,2,5,18]. Extrinsic factors encompass environmental exposure, prolonged visual strain, contact lens use, certain topical (e.g., antiglaucomatous, nonsteroidal anti-inflammatory drugs and all drops containing preservatives such as benzalkonium chloride), and systemic medications (neuroleptics, antidepressants, beta-blockers, antihistamines, diuretics), smoking, poor hydration, and diet [19]. Given this diverse etiology, obtaining a comprehensive medical history is essential for identifying high-risk patients and addressing modifiable contributors to optimize prevention and management [20]. Table 1 provides a summary of key epidemiological studies on DES across different populations, with particular emphasis on pregnancy-related research, highlighting differences in prevalence, diagnostic approaches, and contributing risk factors. Importantly, although the association between hormonal changes and ocular surface homeostasis appears biologically significant, the TFOS DEWS III report does not currently recognize pregnancy as an established risk factor for dry eye disease, and the evidence regarding menopause also remains inconclusive [10]. While menopause has been proposed as a potential contributor to DES, the report emphasizes that definitive large-scale studies are lacking. Taken together, these findings suggest that fluctuations in sex hormones—whether related to pregnancy or menopause—represent plausible mechanisms influencing ocular surface function, yet current evidence is insufficient to establish a direct causal relationship. Accordingly, our review underscores the need for further clinical research while maintaining a cautious interpretation of the available data [21,22]. Table 1 summarizes the main epidemiological studies on dry eye disease across different populations, with particular emphasis on pregnancy-related cohorts, highlighting prevalence rates, diagnostic methods, and associated risk factors.

We hypothesize that pregnancy-related hormonal, metabolic, and immunological changes significantly contribute to ocular surface instability and may trigger or exacerbate dry eye disease. The aim of this review is to provide a comprehensive synthesis of current evidence on DES in pregnancy, focusing on pathophysiology, clinical features, diagnostic strategies, and management approaches tailored to the safety of pregnant women.

## 2. Pathophysiology

Under normal physiological conditions, there is a balance between tear production and evaporation [25]. The tear film comprises three essential layers: lipid, aqueous, and mucin. The lipid layer stabilizes the tear film and reduces evaporation. The aqueous layer provides hydration, removes debris, and transports nutrients [25,26]. The mucin layer, produced by conjunctival goblet cells, ensures adherence of the tear film to the ocular surface [18,27].

Key aspects of DES pathophysiology include tear film dysfunction, hyperosmolarity, inflammation, immune activation, neurogenic dysfunction, and MGD [7,25,28]. Environmental and lifestyle factors such as low humidity, extended screen time, or contact lens use can further stress the ocular surface symptoms [18]. Tear evaporation and reduced production lead to hyperosmolarity, which damages epithelial cells, triggers inflammation, and increases oxidative stress [28,29]. This process results in the release of proinflammatory cytokines (e.g., IL-1, IL-6, TNF-α) and the recruitment of immune cells, perpetuating a cycle of inflammation and tissue damage [28,30]. Corneal nerve dysfunction—manifesting as decreased sensitivity (hypoesthesia) or increased pain perception (neuropathic pain)—also contributes to DES symptoms [18,31]. MGD, a leading cause of evaporative dry eye, is characterized by blockage or altered lipid secretion from the Meibomian glands, compromising the lipid layer and increasing evaporation. Based on the underlying mechanisms, DES is classified as aqueous-deficient dry eye (due to insufficient aqueous tear production, e.g., in Sjögren’s syndrome, and evaporative dry eye (primarily due to MGD or environmental factors) [10,18,31]. Emerging studies underline a strict association between gut microbiota dysbiosis and DES. An abnormal Firmicutes/Bacteroidetes ratio in the gut may lead to a reduction in goblet cells in the conjunctiva and acinar cells in the lacrimal glands, thereby decreasing mucin and tear production [32,33].

In addition to these core mechanisms, a wide range of external and systemic factors contribute to DES pathogenesis. Autoimmune diseases, including Sjögren’s syndrome (where up to 90% of patients develop DES), inflammatory bowel disease, rheumatoid arthritis, systemic lupus erythematosus, and scleroderma, are well-established risk factors [10,34,35]. Other endocrine conditions consistently linked to DES include thyroid disease or disorder, androgen deficiency, polycystic ovarian syndrome, insulin resistance and diabetes [10,36]. Dermatologic and atopic diseases—including rosacea, acne vulgaris, eczema, psoriasis and ocular allergy—further exacerbate ocular surface instability [10,36]. Allergic eye disease and atopic disorders such as asthma often overlap with DES due to shared symptoms such as burning, itching, and tearing [10,37,38]. Chronic pain syndromes (e.g., fibromyalgia, pelvic pain, irritable bowel syndrome, migraine, temporomandibular joint disorder) and psychiatric conditions (depression, anxiety, stress, PTSD) are consistently associated with DES, likely via altered pain perception, somatization, and inflammatory pathways [10,39]. Sleep disorders, particularly obstructive sleep apnea, further contribute through mechanisms such as floppy eyelid syndrome, nocturnal exposure, and systemic low-grade inflammation [10,36]. Ophthalmic comorbidities also play a central role. Almost all ocular surgeries—including refractive and cataract surgery—have been linked to increased DES risk, as have periocular procedures such as blepharoplasty and botulinum toxin injections [10,40,41].

Another significant factor influencing DES is medication—systemic and topical. More than half of the most prescribed drugs have been associated with DES, particularly those with anticholinergic properties (antihistamines, antidepressants, antiarrhythmics, bronchodilators, antiparkinsonian agents). Retinoids (e.g., isotretinoin), anticancer agents, dupilumab, hormone replacement therapy, proton pump inhibitors, and psychostimulants also contribute through various toxic, inflammatory, and metabolic effects [10,42,43,44].

Environmental influences such as extreme temperatures, low humidity, air pollution, and prolonged screen exposure are well-documented triggers, while contact lens wear and certain cosmetic ingredients (e.g., preservatives, prostaglandin analogues, retinoids) further aggravate ocular surface instability [10,45,46]. Finally, nutrition and gut microbiome composition have been implicated, with deficiencies in vitamins A, C, D, B12, and ω-3 fatty acids emerging as modifiable risk factors [10,47].

More recently, COVID-19 has emerged as an additional factor negatively affecting ocular surface health. Both direct viral effects and secondary mechanisms, including systemic inflammation, prolonged mask use, and increased digital screen exposure during and after the pandemic, have been implicated. However, no studies to date have specifically addressed whether pregnancy-related DES has increased since the pandemic, and this remains an area for future research [48,49,50]. In one study of pregnant women with COVID-19, dry eye was reported among the common post-infection symptoms, although no systematic evaluation of DES was performed [50]. Overall, DES arises from the disruption of tear film homeostasis through interacting mechanisms—including tear deficiency, increased evaporation, inflammation, immune and neurosensory dysfunction—further amplified by systemic diseases, hormonal and metabolic changes, medication use, environmental exposures, and, more recently, COVID-19, highlighting its multifactorial and self-perpetuating nature.

## 3. Pregnancy and DES

All the mechanisms of DES pathophysiology contribute to the development of ocular symptoms in pregnancy, positioning pregnant women in a high-risk group [51,52]. In addition to hormonal fluctuations, there are also physiological alterations inherent to pregnancy itself [53]. Pregnant women undergo physiological ocular changes, primarily affecting the cornea and the iridocorneal angle [54,55,56]. Some of these changes may contribute to the exacerbation of dry eye symptoms. Key alterations relevant to DES include a reduction in corneal sensitivity, an increase in corneal thickness (pachymetry) and further risk of corneal edema. The decline in corneal sensitivity may indirectly aggravate ocular discomfort by diminishing corneal reflex sensitivity and reflex tear secretion [16,54,57]. Fluid retention in the corneal stroma may also intensify DES symptoms (decreased vision, foreign body sensation) [55,57]. Moreover, pregnant women often experience contact lens intolerance due to altered corneal curvature and tear film instability [58]. Additionally, pregnancy-related eyelid edema can impair ocular surface hydration and reduce the frequency or completeness of blinking, further contributing to tear film dysfunction [8,16].

While precise prevalence data are limited, studies indicate that DES may affect between 20% and 50% of pregnant women, particularly during the second and third trimesters [37]. Increased risk has been associated with maternal age, pre-existing ocular or systemic conditions (e.g., autoimmune diseases), as well as lifestyle and demographic factors [37]. Table 2 provides an overview of the main clinical studies evaluating the prevalence and characteristics of DES in pregnant women, highlighting differences in populations, diagnostic methods, and symptom severity.

Studies reporting prevalence and clinical characteristics of DES in pregnant women, stratified by trimester and severity of symptoms.

Pregnancy-specific systemic adaptations may further modulate disease expression: maternal immune tolerance, essential for fetal survival, and insulin resistance, which becomes more pronounced in later trimesters, both influence ocular surface homeostasis, though with interindividual variability [59,60]. These factors may increase the risk or severity of dry eye syndrome in certain subgroups of pregnant women. Nevertheless, because fluctuations in sex hormone levels are both universal and profound, our review has focused primarily on endocrine mechanisms, while acknowledging that immunological and metabolic adaptations may modulate disease expression.

Under physiological conditions, sex hormones play a crucial role in maintaining ocular surface homeostasis [8,28,51,61]. Estrogens influence tear film stability by modulating the function of lacrimal and meibomian glands, though their effects may be pro-inflammatory in some contexts [51,61,62]. Additionally, Swiss studies have shown that elevated estrogen levels increase collagenase activity, causing biomechanical changes in the cornea [58,63]. Progesterone is thought to contribute to ocular surface protection, potentially by counteracting estrogen-driven inflammation and supporting epithelial integrity [28,51,61]. Androgens, particularly dehydroepiandrosterone and testosterone, are essential for stimulating meibomian gland secretion and maintaining a stable lipid layer of the tear film [28,51,61]. A deficiency in androgens is strongly associated with MGD and evaporative dry eye [61]. These hormones act on the lacrimal glands, meibomian glands, eyelid tissues, and corneal epithelium through specific hormone receptors, and their balance is vital for tear film quantity and quality [28,51,62].

Fluctuations in sex hormone levels—especially estrogen, progesterone, and androgens—play a central role in DES pathophysiology during pregnancy [8,51,64]. Also perimenopausal and postmenopausal women are generally more prone to DES, particularly due to altered hormonal status [7,8,12,51]. Similar mechanisms are believed to contribute to increase DES symptoms in pregnant women [15,65].

During the first trimester, estrogen levels—particularly estradiol—begin to rise, while estriol levels remain relatively low [66]. Estrogen is primarily produced by the corpus luteum and the developing placenta [67]. The corpus luteum also produces progesterone, which supports embryo implantation, suppresses uterine contractions, and has immunosuppressive effects [68]. Human chorionic gonadotropin rescues the corpus luteum in early pregnancy, sustaining progesterone secretion until placental steroidogenesis takes over [69]. In the second trimester, estriol becomes the dominant estrogen [65,67]. This phase is marked by uterine and mammary gland growth, supported by increased estrogen. The placenta becomes the primary source of progesterone, which relaxes the uterine smooth muscle and prevents premature contractions [15,62]. By the third trimester, estrogen levels rise significantly while progesterone levels decrease [70]. This hormonal shift prepares the body for labor but may also destabilize the tear film, contributing to an increase in DES symptoms [13]. Most pregnant women who experience DES report symptoms after the 30th week of gestation [15,62].

Testosterone levels also rise throughout pregnancy, primarily due to placental and ovarian production, with additional contributions from the fetal adrenal glands and liver [71,72,73]. Despite increased total testosterone, the biologically active (free) fraction remains stable due to a parallel rise in sex hormone-binding globulin [73,74]. However, elevated testosterone levels may still impact Meibomian gland function and ocular surface homeostasis [62].

Prolactin, which increases significantly in the third trimester, is believed to regulate lacrimal gland function, although this remains under investigation [75,76,77,78]. The hormonal milieu of pregnancy undergoes profound changes that influence tear film stability and ocular surface homeostasis [79]. Table 3 summarizes the key hormonal changes during pregnancy and their potential impact on tear film stability, ocular surface homeostasis, and meibomian gland function.

**Figure 2:**
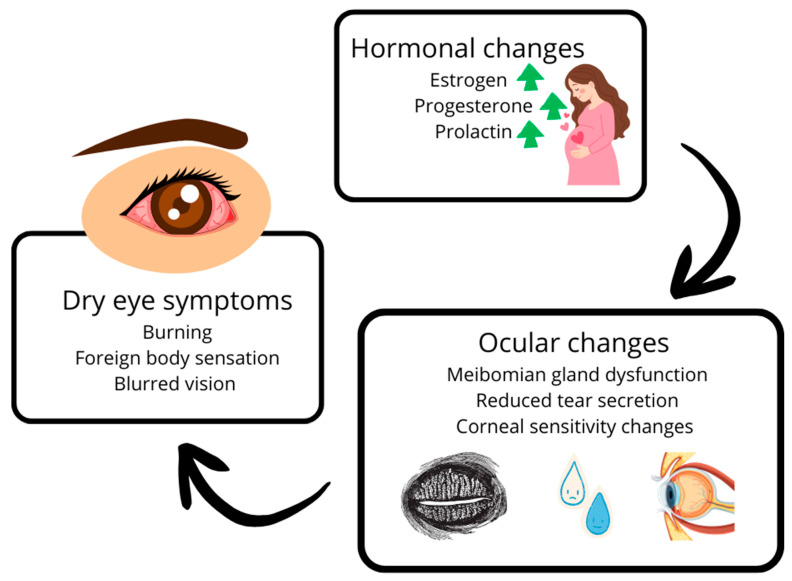
Schematic overview of the relationship between pregnancy-related hormonal changes and dry eye syndrome (DES) pathophysiology. Fluctuations in estrogen, progesterone, and androgens during pregnancy influence tear film stability, meibomian gland function, and corneal homeostasis. Prolactin and other systemic changes, such as insulin resistance and maternal immune tolerance, may further modulate disease severity. Adapted from data summarized in the TFOS DEWS II Sex, Gender, and Hormones Report [8].

The alterations in hormone levels, as previously discussed, can reduce both the quantity and quality of tears [13,52]. Reduced androgen activity is associated with MGD and evaporative dry eye [7,15,62]. The underlying pathophysiological mechanisms are becoming increasingly well understood, and thanks to scientific advancements and ongoing research, we are gaining deeper insights into the causes of the condition, which in turn facilitates prevention and management of its progression and complications [15,28,56,65]. The relationship between pregnancy-related hormonal fluctuations and DES pathophysiology is summarized in Figure 2.

Hormone-related electrolyte imbalances can also affect ocular surface health. Eyelid swelling may interfere with blinking, and corneal changes—such as edema and increased curvature—may lead to tear film instability [58]. Swiss studies have shown that elevated estrogen levels increase collagenase activity, causing biomechanical changes in the cornea [58,63].

Clinical and experimental studies support these mechanisms. Animal studies in pregnant rabbits have shown reduced Schirmer scores, shorter Tear Break-Up Time (TBUT), and greater corneal staining, consistent with hormonal effects on ocular surface stability [80]. Questionnaire-based studies (e.g., SPEED) and large cross-sectional cohorts confirm that many pregnant women report DES symptoms, with higher prevalence and severity in later trimesters. For instance, in an Indian cohort of 428 pregnant women, nearly half experienced mild symptoms, while over 76% reported ocular discomfort [23,81]. Although some studies find no correlation with gestational age, others demonstrate marked symptom worsening in the third trimester. These findings highlight the clinical relevance of DES during pregnancy, with potential implications for maternal comfort and adherence to prenatal care [14,17,23].

Nevertheless, evidence gaps remain. To date, no longitudinal studies have evaluated whether the prevalence of DES in pregnancy has increased over the past decade. Available data are limited to small, cross-sectional studies, precluding reliable temporal comparisons. Similarly, while ocular allergy is a recognized comorbidity that may exacerbate tear film instability and ocular surface inflammation, pregnancy-specific evidence for a causal role is lacking. These factors should therefore be regarded as potential modifiers rather than established determinants of pregnancy-related DES.

## 4. Diagnosis in Pregnancy

In general, the diagnosis of DES during pregnancy follows standard clinical protocols, including detailed medical history, assessment of symptoms, and the use of diagnostic tests [1,28]. However, it is essential to consider pregnancy-specific factors, as some symptoms may overlap with other physiological changes occurring during this time.

Key diagnostic tools include the Schirmer test—measures tear production, TBUT-assesses tear film stability and tear osmolarity tests- evaluate tear concentration [1,82,83]. These methods help confirm the presence and severity of DES [23].

It is crucial for gynecologists to elicit a comprehensive ocular history as part of routine prenatal care [81]. Even in cases of mild symptoms, initial management tailored to gestational age should be considered [84,85]. If symptoms worsen or fail to respond to initial treatment, prompt referral to an ophthalmologist is advised for further evaluation and intervention [85,86].

## 5. Management of DES

The treatment of DES during pregnancy should begin as soon as the first symptoms appear [85]. Due to hormonal changes that occur throughout pregnancy, symptoms may progressively worsen and lead to significant complications [13,28,56]. Close collaboration between the obstetrician and the ophthalmologist is essential to ensure timely and effective initiation and continuation of treatment [85]. Since each trimester is associated with distinct hormonal fluctuations, therapy should be individualized and take into account the patient’s age, overall health, environmental conditions, and, most importantly, gestational age [13,85]. Accordingly, every treatment plan should be tailored to the unique needs of the patient [85]. Management should always prioritize the safety of both the mother and the fetus. The primary treatment goals are to eliminate modifiable risk factors, implement a proper diet, and identify the underlying cause of DES, considering the hormonal, hemodynamic, and metabolic changes occurring during pregnancy [13,85]. In a study conducted by Asiedu K. and colleagues in Ghana examined 201 pregnant women and identified 82 cases of DES, representing approximately 40% of the participants. Among these, 62.2% had unclassified DES, while 18% had evaporative DES, and 12% were diagnosed with aqueous-deficient DES. The remaining cases involved mixed-type disease. This study highlights the complexity of diagnosing and treating DES in pregnant women, as classic forms (evaporative or aqueous-deficient) account for only one-third of all cases [14].

The initial therapeutic approach should focus on patient education—informing them about the condition, available treatments, and expected outcomes [85,86]. Education should also include guidance on lifestyle modifications, such as optimizing environmental conditions, adjusting the diet, discontinuing medications that may worsen symptoms, and practicing proper eyelid hygiene [84,85,86].

Environmental interventions include using portable humidifiers at home and work, avoiding cigarette smoke (both active and passive), limiting screen time, and taking regular breaks from constant reading. Proper hydration is also essential [24,84,85]. For individuals who spend long hours in front of a computer, lowering the monitor below eye level can reduce eyelid aperture and tear evaporation [79,87,88]. Raising the desk chair and consciously increasing blinking frequency can further alleviate symptoms [89,90].

An appropriate diet is another crucial aspect of management [85,91]. Numerous studies and meta-analyses indicate that omega-3 fatty acid intake enhances tear production and reduces ocular surface inflammation [92,93,94]. Omega-3s contain eicosapentaenoic acid (EPA) and docosahexaenoic acid (DHA), both of which possess anti-inflammatory properties that contribute to tear film stability [95]. According to the Institute of Food and Nutrition in Poland, pregnant women should consume an additional 100–200 mg of DHA on top of the standard adult intake of 250 mg/day of combined DHA and EPA. DHA is essential for fetal brain and vision development. As per the Regulation of the Minister of Health (9 August 2019), which established the “Standard of Hospital Nutrition for Pregnant and Postpartum Women—Mother’s Diet” pilot program, fish should be consumed twice weekly. However, due to the risk of contamination, predatory fish (e.g., shark, marlin, swordfish) and certain Baltic fish (e.g., salmon, sprat) should be avoided [96]. The European Food Safety Authority maintains that the benefits of fish consumption (1–4 times per week) during pregnancy outweigh the potential risks. Given the generally low dietary intake of DHA and its critical importance during pregnancy, supplementation is recommended. The Polish Expert Group advises a daily intake of 200 mg of DHA, increasing to 400–600 mg when fish consumption is low. The Polish Society of Gynecology recommends a daily intake of 600 mg, and for women at high risk of preterm labor, at least 1000 mg per day [97]. Another vision-supportive nutrient is vitamin A, which is important for night vision. However, excessive intake can be teratogenic. According to the World Health Organization, the maximum recommended daily intake of vitamin A during pregnancy is 10,000 IU [98,99].

It is also vital to review any medications (systemic and topical) the patient is taking, as beta-blockers, diuretics, and certain antidepressants may induce or aggravate DES symptoms [100,101]. Oral pilocarpine tablets, occasionally prescribed in Sjögren’s syndrome to stimulate exocrine gland secretion, have very limited safety data in pregnancy [102]. No controlled studies in pregnant women are available, and animal studies have reported potential embryotoxic effects at high doses. Regulatory agencies (FDA, EMA) therefore recommend that pilocarpine should not be used during pregnancy unless the potential benefit clearly outweighs the potential risk. In practice, pilocarpine is generally avoided during pregnancy, with management of dry eye focusing instead on safer alternatives such as preservative-free lubricants, punctal occlusion, and environmental modifications. For patients with aqueous-deficient DES, first-line therapy includes over-the-counter preservative-free artificial tears [2,85,86,89,103]. Additionally, preservative-free gels and ointments can be used to lubricate the ocular surface, particularly at bedtime [31,85,86,103].

Patients with MGD require a different approach, focusing on eyelid hygiene—this includes regular cleansing of the eyelid margins, application of warm compresses, and gentle lid massage to improve gland function and tear film quality [1,85,89,104]. In some cases, testing for Demodex mites may be warranted, and if present, tea tree oil-based treatments can be employed [85]. Modern physical therapies, such as pulsed heat therapy, may offer relief for up to 12 months [85,105,106]. If symptoms persist despite first-line therapy, anti-inflammatory medications such as topical glicocorticosteroids, cyclosporine 0.05% or lifitegrast 5% may be considered [85,107,108]. In pregnancy, however, these should be reserved for severe cases and always preceded by ophthalmological evaluation. Similarly, topical antibiotics must be used cautiously and only under strict medical supervision. In exceptional cases, oral antibiotics may be considered [85,109].

When all other treatments are ineffective, therapeutic contact lenses or autologous serum eye drops may be considered [85,110,111,112,113]. Autologous serum eye drops (ASED), derived from the patient’s own blood, contain anti-inflammatory factors that help reduce corneal inflammation [110,111,112,113]. While some early trials found no significant difference between autologous serum and artificial tears [114], more recent randomized controlled studies and meta-analyses demonstrate that ASEDs are more effective—especially in moderate to severe dry eye disease—by improving tear film stability, reducing ocular surface staining, and enhancing patient-reported symptoms [111,115,116]. Contact lens use should be approached with caution in pregnant women with dry eye symptoms [85]. In general, lenses reduce oxygen supply and may cause limbal hypoxia, destabilizing the ocular surface [117,118]. In the third trimester, hormonal and corneal changes further reduce lens tolerance, potentially worsening symptoms and perpetuating the vicious cycle of DES [28]. Temporary discontinuation may be advisable in symptomatic cases [28].

## 6. Prophylaxis of DES in Pregnant Women

Preventing DES during pregnancy requires a proactive approach that combines lifestyle modifications, dietary adjustments, and proper ocular hygiene [114,119]. Given the hormonal fluctuations that can affect tear production and ocular surface stability, early preventive measures are essential to reduce the risk of developing or exacerbating DES symptoms [8,13,51]. The foundation of prophylaxis lies in patient education. Pregnant women should be informed about the symptoms, risk factors, and potential complications associated with DES [1,2,13,51,120]. Proper hydration is critical—not only for overall maternal health but also for maintaining tear film stability [2,119]. Pregnant women should be encouraged to drink adequate fluids throughout the day [14]. Environmental protection is another key element. Patients should avoid exposure to dry environments, air conditioning, and cigarette smoke (both active and passive) [7,85,119]. The use of humidifiers at home or work, as well as sunglasses with UV protection outdoors, can help protect the ocular surface from environmental stressors [14]. Nutritional support also plays a significant role in prevention. A diet rich in omega-3 fatty acids—found in fish, flaxseeds, and walnuts—has been shown to support tear production and reduce inflammation [85,121,122]. In cases where dietary intake is insufficient, DHA supplementation should be considered, with recommendations adjusted according to national guidelines for pregnancy [14]. Maintaining good eyelid hygiene is another effective preventive strategy [85,123]. This includes the use of warm compresses, gentle eyelid cleansing with hypoallergenic products, and regular eyelid massage to promote Meibomian gland function [85,124]. These practices can help maintain a healthy tear film and prevent gland obstruction. For women who spend extended periods in front of screens, the 20-20-20 rule should be followed: every 20 min, look at something 20 feet away for at least 20 s. Adjusting the height of computer monitors to reduce eyelid opening can also help minimize tear evaporation [88,125,126,127,128]. The use of preservative-free artificial tears may be beneficial as a preventive measure to maintain adequate ocular surface hydration. These can be used regularly, even before symptoms become noticeable [129]. Pregnant patients with chronic conditions requiring medications such as diuretics, beta-blockers, neuroleptics or antidepressants—which may contribute to DES —should be evaluated for safer alternatives, assuming that the primary underlying condition allows for such modifications [14].

Ultimately, all pregnant women should be made aware that they are at increased risk of DES. Routine check-ups with both gynecologists and ophthalmologists are recommended, particularly if symptoms aggravate. Collaborative care and a preventive plan established early in pregnancy can help minimize ocular complications and support both maternal and fetal well-being.

## 7. Conclusions

Pregnancy is a unique physiological state in which profound hormonal, metabolic, and immunological changes create a vulnerable environment for the ocular surface. DES is a frequent yet often underrecognized condition in this context, with prevalence estimates ranging from 20% to 50%. Endocrine factors, particularly fluctuations in estrogen and progesterone, represent the most consistent contributors, while additional mechanisms such as insulin resistance and maternal immune tolerance may further modulate disease expression. Although DES in pregnancy is usually mild and self-limiting, it can negatively affect comfort, adherence to prenatal care, and overall quality of life. Obstetricians should recognize DES as a pregnancy-related condition, be prepared to provide initial management in uncomplicated cases, and refer women with more severe or multifactorial disease to ophthalmologists. Increased awareness, early recognition, and individualized care are essential for optimizing maternal ocular health and preventing avoidable complications. Future research should further elucidate the underlying pathophysiological mechanisms and establish safe, effective treatment strategies tailored for pregnant women.

## Figures and Tables

**Table 1:** Epidemiological studies on dry eye syndrome (DES).

No.	Study	Population Studied	Sample Size (n)	Prevalence (%)	Diagnostic Method	Notes
1	Moss et al. (2000) [7]	USA, general population (40–97 years)	3722	14.4	Questionnaire + clinical signs	Prevalence increases with age
2	Schaumberg et al. (2003) [11]	USA, postmenopausal women	25,665	7.8 diagnosed	Self-reported symptoms	Higher prevalence in women
3	Han et al. (2011) [3]	Korea, adults aged 20+	16,824	8.0	Questionnaire + interview	Industrial environments increase risk
4	Asiedu et al. (2021) [14]	Ghana, pregnant women	201	40.8	TBUT, Schirmer, OSDI	High prevalence in pregnancy
5	Anantharaman et al. (2023) [23]	India, pregnant women	428	76 subjective	SPEED questionnaire	Symptoms intensify in third trimester
6	Sahai & Malik (2005) [4]	India, general population	200	29.0	TBUT, fluorescein, Schirmer	Related to dry air and screen use
7	Stapleton et al. (2017) [2]	Systematic review (various countries)	—	5–50	Various	High variability by region
8	Farrand et al. (2017) [5]	USA, general adult population	75,000	6.8	ICD-9 code (insurance database)	Large cohort using administrative data
9	Skare et al. (2012) [15]	Brazil, pregnant vs. non-pregnant women	90	18.9	Questionnaire	Symptoms more common in pregnant women
10	Gayton (2009) [24]	Expert review	—	5–30	—	Emphasizes hormonal and environmental influence

Abbreviations: TBUT, tear break-up time; OSDI, Ocular Surface Disease Index; SPEED, Standard Patient Evaluation of Eye Dryness.

**Table 2:** DES in pregnancy—Summary of Literature-Based Studies.

Authors	Number of Participants	Number of Pregnant Women	Percentage with DES	Comments
Asiedu et al. (2021) [14]	201	82	40.8%	Cross-sectional study; higher prevalence in 3rd trimester
Anantharaman et al. (2023) [23]	428	203	48.5%	SPEED questionnaire; symptoms more severe later
Sarikaya & Acet (2022) [17]	3 groups: P16 = 49; P32 = 46; NP = 51	95	33–40% in pregnancies	Compared with controls; meibomian gland

**Table 3:** Hormonal changes during pregnancy and their impact on the tear film.

Hormone	Change During Pregnancy	Action	Effect on Tear Film	Clinical Manifestation
Estrogen [21,70]	Significant increase (estriol especially II & III trimester)	1. Inhibits lipogenesis in Meibomian glands (5α-reductase) 2. Alters cytokine expression	Decrease lipid layer → increased evaporation	Destabilization of tear film
Progesterone [21,31]	Steady increase (especially II and III trimester)	1. Immunosuppressive effect 2. Reduces pro-inflammatory cytokines 3. Supports epithelial integrity	Potentially protective, reduces inflammation	Mixed, potentially protective
Prolactin [21,31]	Gradual increase, peak in III trimester	1. May enhance lacrimal gland function (prolactin receptors)	Increase in aqueous tear production	Possibly protective, limited evidence
Androgens [21,31]	Relative decrease	1. Stimulates glands 2. Stimulates lacrimal glands	Reduces lipid and mucin secretion	Meibomian Gland Dysfunction, DES
β-hCG [21,31]	Rapid increase in early pregnancy	Stimulates corpus luteum to maintain estrogens and progesterone	Indirect influence on estrogen and progesterone level	Contributes to hormonal imbalance

## Data Availability

Not applicable.

## References

[1] Craig J.P., Nichols K.K., Akpek E.K., Caffery B., Dua H.S., Joo C.-K., Liu Z., Nelson J.D., Nichols J.J., Tsubota K. (2017). TFOS DEWS II Definition and Classification Report. Ocul. Surf..

[2] Stapleton F., Alves M., Bunya V.Y., Jalbert I., Lekhanont K., Malet F., Na K.-S., Schaumberg D., Uchino M., Vehof J. (2017). TFOS DEWS II Epidemiology Report. Ocul. Surf..

[3] Han S.B., Hyon J.Y., Woo S.J., Lee J.J., Kim T.H., Kim K.W. (2011). Prevalence of dry eye disease in an elderly Korean population. Arch. Ophthalmol..

[4] Sahai A., Malik P. (2005). Dry eye: Prevalence and attributable risk factors in a hospital-based population. Indian J. Ophthalmol..

[5] Farrand K.F., Fridman M., Stillman I.Ö., Schaumberg D.A. (2017). Prevalence of Diagnosed Dry Eye Disease in theUnited States Among Adults Aged 18 Years and Older. Arch. Ophthalmol..

[6] Barabino S. (2022). Is dry eye disease the same in young and old patients? A narrative review of the literature. BMC Ophthalmol..

[7] Moss S.E., Klein R., Klein B.E.K. (2000). Prevalence of and risk factors for dry eye. Arch. Ophthalmol..

[8] Sullivan D.A., Rocha E.M., Aragona P., Clayton J.A., Ding J., Golebiowski B., Hampel U., McDermott A.M., Schaumberg D.A., Srinivasan S. (2017). TFOS DEWS II Sex, Gender, and Hormones Report. Ocul. Surf..

[9] Bhatt K., Singh S., Singh K., Kumar S., Dwivedi K. (2023). Prevalence of dry eye, its categorization (Dry Eye Workshop II), and pathological correlation: A tertiary care study. Indian J. Ophthalmol..

[10] Stapleton F., Argüeso P., Asbell P., Azar D., Bosworth C., Chen W., Ciolino J.B., Craig J.P., Gallar J., Galor A. (2025). TFOS DEWS III: Digest. Arch. Ophthalmol..

[11] Schaumberg D.A., Sullivan D.A., Buring J.E., Dana M.R. (2003). Prevalence of dry eye syndrome among US women. Arch. Ophthalmol..

[12] Versura P., Campos E.C. (2005). Menopause and dry eye. A possible relationship. Gynecol. Endocrinol..

[13] Versura P., Giannaccare G., Campos E.C. (2014). Sex-steroid imbalance in females and dry eye. Curr. Eye Res..

[14] Asiedu K., Kyei S., Adanusa M., Ephraim R.K.D., Animful S., Ali-Baya S.K., Akorsah B., Sekyere M.A. (2021). Dry eye, its clinical subtypes and associated factors in healthy pregnancy: A cross-sectional study. PLoS ONE.

[15] Skare T.L., Gehlen M.L., Silveira D.M.G., Uema M.M. (2012). Lacrimal dysfunction and pregnancy. Rev. Bras. Hematol. Hemoter..

[16] Naderan M. (2018). Ocular changes during pregnancy. J. Curr. Ophthalmol..

[17] Sarikaya S., Acet Y. (2022). The effect of pregnancy on meibomian gland, tear film, cornea and anterior segment parameters. Photodiagnosis Photodyn. Ther..

[18] Huang R., Su C., Fang L., Lu J., Chen J., Ding Y. (2022). Dry eye syndrome: Comprehensive etiologies and recent clinical trials. Int. Ophthalmol..

[19] Zahra A.I.Y.H. (2021). Dry eye syndrome risk factors: A systemic review. Saudi J Ophthalmol..

[20] Britten-Jones A.C., Wang M.T.M., Samuels I., Jennings C., Stapleton F., Craig J.P. (2024). Epidemiology and Risk Factors of Dry Eye Disease: Considerations for Clinical Management. Medicina.

[21] Wolffsohn J.S., Benítez-Del-Castillo J.M., Loya-Garcia D., Inomata T., Iyer G., Liang L., Pult H., Sabater A.L., Starr C.E., Vehof J. (2025). TFOS DEWS III: Diagnostic Methodology. Arch. Ophthalmol..

[22] Jones L., Craig J.P., Markoulli M., Karpecki P., Akpek E.K., Basu S., Bitton E., Chen W., Dhaliwal D.K., Dogru M. (2025). TFOS DEWS III: Management and Therapy. Arch. Ophthalmol..

[23] Anantharaman D., Radhakrishnan A., Anantharaman V. (2023). Subjective Dry Eye Symptoms in Pregnant Women—A SPEED Survey. J. Pregnancy.

[24] Gayton J. (2009). Etiology, prevalence, and treatment of dry eye disease. Clin. Ophthalmol..

[25] Willcox M.D.P., Argüeso P., Georgiev G.A., Holopainen J.M., Laurie G.W., Millar T.J., Papas E.B., Rolland J.P., Schmidt T.A., Stahl U. (2017). TFOS DEWS II Tear Film Report. Ocul. Surf..

[26] Mathers W. (2004). Evaporation from the ocular surface. Exp. Eye Res..

[27] Hodges R.R., Dartt D.A. (2013). Tear film mucins: Front line defenders of the ocular surface; comparison with airway and gastrointestinal tract mucins. Exp. Eye Res..

[28] Bron A.J., De Paiva C.S., Chauhan S.K., Bonini S., Gabison E.E., Jain S., Knop E., Markoulli M., Ogawa Y., Perez V. (2017). TFOS DEWS II pathophysiology report. Ocul. Surf..

[29] Bu J., Liu Y., Zhang R., Lin S., Zhuang J., Sun L.E., Zhang L., He H., Zong R., Wu Y. (2024). Potential New Target for Dry Eye Disease—Oxidative Stress. Antioxidants.

[30] Yoon K.-C., Jeong I.-Y., Park Y.-G., Yang S.-Y. (2007). Interleukin-6 and Tumor Necrosis Factor-α Levels in Tears of Patients with Dry Eye Syndrome. Cornea.

[31] (2007). The definition and classification of dry eye disease: Report of the Definition and Classification Subcommittee of the International Dry Eye WorkShop (2007). Ocul. Surf..

[32] Moon J., Yoon C.H., Choi S.H., Kim M.K. (2020). Can Gut Microbiota Affect Dry Eye Syndrome?. Int. J. Mol. Sci..

[33] Trujillo-Vargas C.M., Schaefer L., Alam J., Pflugfelder S.C., Britton R.A., De Paiva C.S. (2020). The gut-eye-lacrimal gland-microbiome axis in Sjögren Syndrome. Ocul. Surf..

[34] Turk M.A., Hayworth J.L., Nevskaya T., Pope J.E. (2020). Ocular Manifestations in Rheumatoid Arthritis, Connective Tissue Disease, and Vasculitis: A Systematic Review and Metaanalysis. J. Rheumatol..

[35] Ko Y.-T., Wu Y.-M., Wu H.-L., Lai S.-C., Dai Y.-X., Chen T.-J., Cherng Y.-G., Tai Y.-H., Kao C.-Y. (2023). Inflammatory bowel disease and the associated risk of dry eye and ocular surface injury: A nationwide matched cohort study. BMC Ophthalmol..

[36] Qian L., Wei W. (2022). Identified risk factors for dry eye syndrome: A systematic review and meta-analysis. PLoS ONE.

[37] Akasaki Y., Inomata T., Sung J., Nakamura M., Kitazawa K., Shih K.C., Adachi T., Okumura Y., Fujio K., Nagino K. (2022). Prevalence of Comorbidity between Dry Eye and Allergic Conjunctivitis: A Systematic Review and Meta-Analysis. J. Clin. Med..

[38] Huang Q., Zheng Y., Zhang C., Wang W., Liao T., Xiao X., Wang J., Wang J. (2021). Association between asthma and dry eye disease: A meta-analysis based on observational studies. BMJ Open.

[39] Basilious A., Xu C.Y., Malvankar-Mehta M.S. (2021). Dry eye disease and psychiatric disorders: A systematic review and meta-analysis. Eur. J. Ophthalmol..

[40] Miura M., Inomata T., Nakamura M., Sung J., Nagino K., Midorikawa-Inomata A., Zhu J., Fujimoto K., Okumura Y., Fujio K. (2022). Prevalence and Characteristics of Dry Eye Disease After Cataract Surgery: A Systematic Review and Meta-Analysis. Ophthalmol. Ther..

[41] Gao M., Xia F., Wang P., Feng Z., Wang X. (2024). Influence of serial intravitreal injections on measures of dry eye: A systemic review and meta-analysis. Contact Lens Anterior Eye.

[42] Wolpert L.E., Snieder H., Jansonius N.M., Utheim T.P., Hammond C.J., Vehof J. (2021). Medication use and dry eye symptoms: A large, hypothesis-free, population-based study in the Netherlands. Ocul. Surf..

[43] Hampel U., Schuster A.K., Nickels S., Schulz A., Lackner K.J., Münzel T., Wild P.S., Beutel M., Schmidtmann I., Pfeiffer N. (2020). Schirmer test results: Are they associated with topical or systemic medication?. Ocul. Surf..

[44] Katipolu Z., Abay R.N. (2023). The relationship between dry eye disease and anticholinergic burden. Eye.

[45] Monge-Carmona R., Caro-Magdaleno M., Sánchez-González M.C. (2024). Association between the use of prostaglandin analogues and ocular surface disease: A systematic review. Eye.

[46] Alves M., Asbell P., Dogru M., Giannaccare G., Grau A., Gregory D., Kim D.H., Marini M.C., Ngo W., Nowinska A. (2023). TFOS Lifestyle Report: Impact of environmental conditions on the ocular surface. Ocul. Surf..

[47] Markoulli M., Ahmad S., Arcot J., Arita R., Benitez-Del-Castillo J., Caffery B., Downie L.E., Edwards K., Flanagan J., Labetoulle M. (2023). TFOS Lifestyle: Impact of nutrition on the ocular surface. Ocul. Surf..

[48] Ji H., Yang Y., Lu Y., Kong X., Yang G., Liu J., Yang Y., Wang X., Ma X. (2023). Prevalence of dry eye during the COVID-19 pandemic: A systematic review and meta-analysis. PLoS ONE.

[49] Szkodny D., Wylęgała A., Chlasta-Twardzik E., Wylęgała E. (2022). The Ocular Surface Symptoms and Tear Film Parameters during and after COVID-19 Infection. J. Clin. Med..

[50] Khalil E.M., Madney Y.M., Hassan M., Fahmy A.M., Alshammari S.O., Alshammari Q.A., Abou-Taleb H.A., Taha A.A., Elgendy M.O., Ali H.A.A. (2024). Maternal and Fetal Outcome of COVID-19 Infection among Pregnant Women. Medicina.

[51] Truong S., Cole N., Stapleton F., Golebiowski B. (2014). Sex hormones and the dry eye. Clin. Exp. Optom..

[52] Sullivan D.A., Wickham L.A., Rocha E.M., Kelleher R.S., Da Silveira L.A., Toda I. (1998). Influence of gender, sex steroid hormones, and the hypothalamic-pituitary axis on the structure and function of the lacrimal gland. Adv. Exp. Med. Biol..

[53] Chawla S., Chaudhary T., Aggarwal S., Maiti G.D., Jaiswal K., Yadav J. (2013). Ophthalmic considerations in pregnancy. Med. J. Armed Forces India.

[54] Goldich Y., Cooper M., Barkana Y., Tovbin J., Ovadia K.L., Avni I., Zadok D. (2014). Ocular anterior segment changes in pregnancy. J. Cataract. Refract. Surg..

[55] Uzun I., Mutaf Ç., Reyhan A.H., Yüksekyayla F., Colak E., Yolaçan M. (2025). Pregnancy-induced ocular changes: Impacts on intraocular pressure, the cornea, and the anterior chamber. BMC Ophthalmol..

[56] Marcos-Figueiredo P., Marcos-Figueiredo A., Menéres P., Braga J. (2017). Ocular Changes During Pregnancy. Rev. Bras. Hematol. Hemoter..

[57] Wang C., Li A.-L., Pang Y., Lei Y.-Q., Yu L. (2017). Changes in intraocular pressure and central corneal thickness during pregnancy: A systematic review and Meta-analysis. Int. J. Ophthalmol..

[58] Park S.B., Lindahl K.J., Temnycky G.O., Aquavella J.V. (1992). The effect of pregnancy on corneal curvature. CLAO J..

[59] Wang J., Han T., Zhu X. (2024). Role of maternal-fetal immune tolerance in the establishment and maintenance of pregnancy. Chin. Med. J..

[60] Lima-Fontes M., Barata P., Falcão M., Carneiro Â. (2020). Ocular findings in metabolic syndrome: A review. Porto Biomed. J..

[61] Sullivan D.A., Yamagami H., Liu M., Steagall R.J., Schirra F., Suzuki T., Krenzer K.L., Cermak J.M., Sullivan R.M., Richards S.M. (2002). Sex steroids, the meibomian gland and evaporative dry eye. Adv. Exp. Med. Biol..

[62] Sullivan D.A., Schaumberg D.A., Suzuki T., Schirra F., Liu M., Richards S., Sullivan R.M., Dana M.R., Sullivan B.D. (2002). Sex steroids, meibomian gland dysfunction and evaporative dry eye in Sjögren’s syndrome. Lupus.

[63] Fatt I., Harris M.G. (1973). Refractive index of the cornea as a function of its thickness. Optom. Vis. Sci..

[64] Mostafa S., Seamon V., Azzarolo A.M. (2012). Influence of sex hormones and genetic predisposition in Sjögren’s syndrome: A new clue to the immunopathogenesis of dry eye disease. Exp. Eye Res..

[65] Anton N., Doroftei B., Ilie O.-D., Ciuntu R.-E., Bogdănici C.M., Nechita-Dumitriu I. (2021). A Narrative Review of the Complex Relationship between Pregnancy and Eye Changes. Diagnostics.

[66] Parisi F., Fenizia C., Introini A., Zavatta A., Scaccabarozzi C., Biasin M., Savasi V. (2023). The pathophysiological role of estrogens in the initial stages of pregnancy: Molecular mechanisms and clinical implications for pregnancy outcome from the periconceptional period to end of the first trimester. Hum. Reprod. Updat..

[67] Napso T., Yong H.E.J., Lopez-Tello J., Sferruzzi-Perri A.N. (2018). The Role of Placental Hormones in Mediating Maternal Adaptations to Support Pregnancy and Lactation. Front. Physiol..

[68] Bulletti C., Bulletti F.M., Sciorio R., Guido M. (2022). Progesterone: The Key Factor of the Beginning of Life. Int. J. Mol. Sci..

[69] Cole L.A. (2010). Biological functions of hCG and hCG-related molecules. Reprod. Biol. Endocrinol..

[70] Albrecht E.D., Pepe G.J. (2010). Estrogen regulation of placental angiogenesis and fetal ovarian development during primate pregnancy. Int. J. Dev. Biol..

[71] Tulchinsky D., Hobel C.J., Yeager E., Marshall J.R. (1972). Plasma estrone, estradiol, estriol, progesterone, and 17-hydroxyprogesterone in human pregnancy. I. Normal pregnancy. Am. J. Obstet. Gynecol..

[72] Nabi G., Aziz T., Amin M., Khan A.A. (2014). Effect of Fetal Sex on Total Levels of Maternal Serum Testosterone. J. Biol. Life Sci..

[73] Dupouy J.P., Coffigny H., Magre S. (1975). Maternal and foetal corticosterone levels during late pregnancy in rats. J. Endocrinol..

[74] Schuijt M.P., Sweep C.G.J., Van der Steen R., Olthaar A.J., Stikkelbroeck N.M.M.L., Ross H.A., Van Herwaarden A.E. (2019). Validity of free testosterone calculation in pregnant women. Endocr. Connect..

[75] McClellan K.A., Robertson F.G., Kindblom J., Wennbo H., Törnell J., Bouchard B., Kelly P.A., Ormandy C.J. (2001). Investigation of the Role of Prolactin in the Development and Function of the Lacrimal and Harderian Glands Using Genetically Modified Mice. Investig. Ophthalmol. Vis. Sci..

[76] Barbosa A.P., de Oliveira F.R., da Rocha F.J., Muglia V.F., Rocha E.M. (2021). Lacrimal gland atrophy and dry eye related to isotretinoin, androgen, and prolactin: Differential diagnosis for Sjögren’s syndrome. Arq. Bras. Oftalmol..

[77] Jüngert K., Paulsen F., Jacobi C., Horwath-Winter J., Garreis F. (2022). Prolactin Inducible Protein, but Not Prolactin, Is Present in Human Tears, Is Involved in Tear Film Quality, and Influences Evaporative Dry Eye Disease. Front. Med..

[78] Kyei S., Ephraim R.K.D., Animful S., Adanusa M., Ali-Baya S.K., Akorsah B., Sekyere M.A., Asiedu K. (2020). Impact of Serum Prolactin and Testosterone Levels on the Clinical Parameters of Dry Eye in Pregnant Women. J. Ophthalmol..

[79] Argilés M., Cardona G., Pérez-Cabré E., Rodríguez M. (2015). Blink Rate and Incomplete Blinks in Six Different Controlled Hard-Copy and Electronic Reading Conditions. Investig. Opthalmology Vis. Sci..

[80] Zhou L., Wei R., Zhao P., Koh S.K., Beuerman R.W., Ding C. (2013). Proteomic analysis revealed the altered tear protein profile in a rabbit model of Sjögren’s syndrome-associated dry eye. Proteomics.

[81] Gomes J.A.P., Azar D.T., Baudouin C., Efron N., Hirayama M., Horwath-Winter J., Kim T., Mehta J.S., Messmer E.M., Pepose J.S. (2017). TFOS DEWS II iatrogenic report. Ocul. Surf..

[82] Baudouin C., Messmer E.M., Aragona P., Geerling G., Akova Y.A., Benítez-Del-Castillo J., Boboridis K.G., Merayo-Lloves J., Rolando M., Labetoulle M. (2016). Revisiting the vicious circle of dry eye disease: A focus on the pathophysiology of meibomian gland dysfunction. Br. J. Ophthalmol..

[83] Sullivan B.D., Crews L.A., Messmer E.M., Foulks G.N., Nichols K.K., Baenninger P., Geerling G., Figueiredo F., Lemp M.A. (2012). Correlations between commonly used objective signs and symptoms for the diagnosis of dry eye disease: Clinical implications. Acta Ophthalmol..

[84] Wolffsohn J.S., Arita R., Chalmers R., Djalilian A., Dogru M., Dumbleton K., Gupta P.K., Karpecki P., Lazreg S., Pult H. (2017). TFOS DEWS II Diagnostic Methodology report. Ocul. Surf..

[85] Jones L., Downie L.E., Korb D., Benitez-Del-Castillo J.M., Dana R., Deng S.X., Dong P.N., Geerling G., Hida R.Y., Liu Y. (2017). TFOS DEWS II Management and Therapy Report. Ocul. Surf..

[86] Messmer E.M. (2015). The pathophysiology, diagnosis, and treatment of dry eye disease. Dtsch. Arztebl. Int..

[87] Portello J.K., Rosenfield M., Chu C.A. (2013). Blink rate, incomplete blinks and computer vision syndrome. Optom. Vis. Sci..

[88] Rosenfield M. (2011). Computer vision syndrome: A review of ocular causes and potential treatments. Ophthalmic Physiol. Opt..

[89] Justin Kwan O.D. (2017). A comprehensive look at dry eye therapy. Rev. Optom..

[90] Amescua G., Ahmad S., Cheung A.Y., Choi D.S., Jhanji V., Lin A., Mian S.I., Rhee M.K., Viriya E.T., Mah F.S. (2024). Dry Eye Syndrome Preferred Practice Pattern^®^. Ophthalmology.

[91] Rashid S., Jin Y., Ecoiffier T., Barabino S., Schaumberg D.A., Dana M.R. (2008). Topical omega-3 and omega-6 fatty acids for treatment of dry eye. Arch. Ophthalmol..

[92] Kaya A., Aksoy Y. (2016). Omega-3 fatty acid supplementation improves dry eye symptoms in patients with glaucoma: Results of a prospective multicenter study. Clin. Ophthalmol..

[93] Liu A., Ji J. (2014). Omega-3 essential fatty acids therapy for dry eye syndrome: A meta-analysis of randomized controlled studies. Med. Sci. Monit..

[94] Kawakita T., Kawabata F., Tsuji T., Kawashima M., Shimmura S., Tsubota K. (2013). Effects of dietary supplementation with fish oil on dry eye syndrome subjects: Randomized controlled trial. Biomed. Res..

[95] McCusker M.M., Durrani K., Payette M.J., Suchecki J. (2016). An eye on nutrition: The role of vitamins, essential fatty acids, and antioxidants in age-related macular degeneration, dry eye syndrome, and cataract. Clin. Dermatol..

[96] Wierzejska R., Jarosz M., Wojda B., Siuba-Strzelińska M. (2018). Dietary intake of DHA during pregnancy: A significant gap between the actual intake and current nutritional recommendations. Rocz. Panstw. Zakl. Hig..

[97] Dębski R., Karowicz-Bilińska A., Oszukowski P., Paszkowski T., Spaczyński M. (2014). Rekomendacje Polskiego Towarzystwa Ginekologicznego dotyczące zastosowania suplementacji kwasem dokozaheksaenowym w profilaktyce poroduprzedwczesnego. Ginekol. Pol..

[98] Penniston K.L., Tanumihardjo S.A. (2006). The acute and chronic toxic effects of vitamin A. Am. J. Clin. Nutr..

[99] Sommer A. (2008). Vitamin a deficiency and clinical disease: An historical overview. J. Nutr..

[100] Fraunfelder F.T., Sciubba J.J., Mathers W.D. (2012). The role of medications in causing dry eye. J. Ophthalmol..

[101] Guo M., Diaz G.M., Yu Y., Patel C.A., Farrar J.T., Asbell P.A., Ying G.-S. (2024). Association between systemic medication use and severity of dry eye signs and symptoms in the DRy eye assessment and management (DREAM) study. Ocul. Surf..

[102] Vivino F.B., Al-Hashimi I., Khan Z., LeVeque F.G., Salisbury P.L., Tran-Johnson T.K., Muscoplat C.C., Trivedi M., Goldlust B., Gallagher S.C. (1999). Pilocarpine tablets for the treatment of dry mouth and dry eye symptoms in patients with Sjögren syndrome: A randomized, placebo-controlled, fixed-dose, multicenter trial. Arch. Intern. Med..

[103] Walsh K., Jones L. (2019). The use of preservatives in dry eye drops. Clin. Ophthalmol..

[104] Lindstrom R., Holland E., Lane S., Raizman M., Reilly C. (2008). Double-masked comparison of ketorolac tromethamine 0.4% versus nepafenac sodium 0.1% for postoperative healing rates and pain control in eyes undergoing surface ablation. Cornea.

[105] Blackie C.A., Carlson A.N., Korb D.R. (2015). Treatment for meibomian gland dysfunction and dry eye symptoms with a single-dose vectored thermal pulsation: A review. Curr. Opin. Ophthalmol..

[106] Blackie C.A., Murakami D., Donnenfeld E., Oliff H.S. (2024). Vectored Thermal Pulsation as a Treatment for Meibomian Gland Dysfunction: A Review Spanning 15 Years. Ophthalmol. Ther..

[107] Hovanesian J.A., Nichols K.K., Jackson M., Katz J., Chan A., Glassberg M.B., Sloesen B., Korves C., Nguyen C., Syntosi A. (2021). Real-World Experience with Lifitegrast Ophthalmic Solution (Xiidra) in the US and Canada: Retrospective Study of Patient Characteristics, Treatment Patterns, and Clinical Effectiveness in 600 Patients with Dry Eye Disease. Clin. Ophthalmol..

[108] Rhee M.K., Mah F.S. (2017). Clinical utility of cyclosporine (CsA) ophthalmic emulsion 0.05% for symptomatic relief in people with chronic dry eye: A review of the literature. Clin. Ophthalmol..

[109] Haber S.L., Benson V., Buckway C.J., Gonzales J.M., Romanet D., Scholes B. (2019). Lifitegrast: A novel drug for patients with dry eye disease. Ther. Adv. Ophthalmol..

[110] Geerling G., Hartwig D. (2002). Autologous serum-eye-drops for ocular surface disorders. A literature review and recommendations for their application. Der Ophthalmol..

[111] Geerling G., Maclennan S., Hartwig D. (2004). Autologous serum eye drops for ocular surface disorders. Br. J. Ophthalmol..

[112] Cui D., Li G., Akpek E.K. (2021). Autologous serum eye drops for ocular surface disorders. Curr. Opin. Allergy Clin. Immunol..

[113] García-Martín E., Pernía-Lpez S., Jiménez R.R., García-Valcárcel B., Martínez-Ortega P.A., Sanjurjo-Saez M. (2018). The use of autologous serum eye drops for the treatment of ocular surface disorders. Eur. J. Hosp. Pharm..

[114] Ness T., Paulus W. (2012). Ophthalmic agents during pregnancy and breastfeeding. Der Ophthalmol..

[115] Pan Q., Angelina A., Marrone M., Stark W.J., Akpek E.K. (2017). Autologous serum eye drops for dry eye. J. Musculoskelet. Neuronal Interact..

[116] He C.-Z., Zeng Z.-J., Liu J.Q., Qiu Q., He Y. (2024). Autologous serum eye drops for patients with dry eye disease: A systematic review and meta-analysis of randomized controlled trials. Front. Med..

[117] Brennan N.A. (2005). Corneal oxygenation during contact lens wear: Comparison of diffusion and EOP-based flux models. Clin. Exp. Optom..

[118] Sweeney D.F. (2013). Have Silicone Hydrogel Lenses Eliminated Hypoxia?. Eye Contact Lens Sci. Clin. Pr..

[119] Craig J.P., Nelson J.D., Azar D.T., Belmonte C., Bron A.J., Chauhan S.K., De Paiva C.S., Gomes J.A.P., Hammitt K.M., Jones L. (2017). TFOS DEWS II Report Executive Summary. Ocul. Surf..

[120] Sutphin J.E., Ying G.-S., Bunya V.Y., Yu Y., Lin M.C., McWilliams K., Schmucker E., Kuklinski E.J., Asbell P.A., Maguire M.G. (2021). Correlation of Measures From the OCULUS Keratograph and Clinical Assessments of Dry Eye Disease in the Dry Eye Assessment and Management Study. Cornea.

[121] Miljanović B., Trivedi K.A., Dana M.R., Gilbard J.P., Buring J.E., Schaumberg D.A. (2005). Relation between dietary n-3 and n-6 fatty acids and clinically diagnosed dry eye syndrome in women. Am. J. Clin. Nutr..

[122] Barabino S., Rolando M., Camicione P., Ravera G., Zanardi S., Giuffrida S., Calabria G. (2003). Systemic Linoleic and -Linolenic Acid Therapy in Dry Eye Syndrome With an Inflammatory Component. Cornea.

[123] Chen F., Wang J., Chen W., Shen M., Xu S., Lu F. (2010). Upper Punctal Occlusion versus Lower Punctal Occlusion in Dry Eye. Investig. Opthalmology Vis. Sci..

[124] Nikopoulos K., Schrauwen I., Simon M., Collin R.W.J., Veckeneer M., Keymolen K., Van Camp G., Cremers F.P.M., Van Den Born L.I. (2011). Autosomal Recessive Stickler Syndrome in Two Families Is Caused by Mutations in the COL9A1 Gene. Investig. Opthalmology Vis. Sci..

[125] Talens-Estarelles C., Cerviño A., García-Lázaro S., Fogelton A., Sheppard A., Wolffsohn J.S. (2022). The effects of breaks on digital eye strain, dry eye and binocular vision: Testing the 20-20-20 rule. Contact Lens Anterior Eye.

[126] Sheppard A.L., Wolffsohn J.S. (2018). Digital eye strain: Prevalence, measurement and amelioration. BMJ Open Ophthalmol..

[127] Portello J.K., Rosenfield M., Bababekova Y., Estrada J.M., Leon A. (2012). Computer-related visual symptoms in office workers. Ophthalmic Physiol. Opt..

[128] Chu C.A., Rosenfield M., Portello J.K. (2014). Blink patterns: Reading from a computer screen versus hard copy. Optom. Vis. Sci..

[129] Semp D.A., Beeson D., Sheppard A.L., Dutta D., Wolffsohn J.S. (2023). Artificial Tears: A Systematic Review. Clin. Optom..

